# Engineering of cellobiose phosphorylase for the defined synthesis of cellotriose

**DOI:** 10.1007/s00253-020-10820-8

**Published:** 2020-08-17

**Authors:** Zorica Ubiparip, David Sáez Moreno, Koen Beerens, Tom Desmet

**Affiliations:** Centre for Synthetic Biology (CSB), Department of Biotechnology, Ghent University, Coupure Links 653, 9000 Ghent, Belgium

**Keywords:** Enzyme engineering, Cellotriose synthesis, Prebiotic, Cellobiose phosphorylase

## Abstract

Cellodextrins are non-digestible oligosaccharides that have attracted interest from the food industry as potential prebiotics. They are typically produced through the partial hydrolysis of cellulose, resulting in a complex mixture of oligosaccharides with a varying degree of polymerisation (DP). Here, we explore the defined synthesis of cellotriose as product since this oligosaccharide is believed to be the most potent prebiotic in the mixture. To that end, the cellobiose phosphorylase (CBP) from *Cellulomonas uda* and the cellodextrin phosphorylase (CDP) from *Clostridium cellulosi* were evaluated as biocatalysts, starting from cellobiose and α-d-glucose 1-phosphate as acceptor and donor substrate, respectively. The CDP enzyme was shown to rapidly elongate the chains towards higher DPs, even after extensive mutagenesis. In contrast, an optimised variant of CBP was found to convert cellobiose to cellotriose with a molar yield of 73%. The share of cellotriose within the final soluble cellodextrin mixture (DP2-5) was 82%, resulting in a cellotriose product with the highest purity reported to date. Interestingly, the reaction could even be initiated from glucose as acceptor substrate, which should further decrease the production costs.

Key points

• *Cellobiose phosphorylase is engineered for the production of cellotriose.*

• *Cellotriose is synthesised with the highest purity and yield to date.*

• *Both cellobiose and glucose can be used as acceptor for cellotriose production.*

**Electronic supplementary material:**

The online version of this article (10.1007/s00253-020-10820-8) contains supplementary material, which is available to authorized users.

## Introduction

Oligosaccharides have continuously growing applications in the food, feed and pharmaceutical industries (Han et al. 2012; Meyer et al. 2015; Martins et al. 2019). In particular, non-digestible oligosaccharides can serve as prebiotics that promote immune-modulatory health effects by influencing the gut microbiome composition (Panesar and Bali 2015; Holscher 2017; Wu et al. 2017). Examples of prebiotics that are already well established on the market include fructooligosaccharides (FOS), galactooligosaccharides (GOS), soybean-derived oligosaccharides (SOS) and (arabino)xylooligosaccharides (XOS/AXOS) (Gibson 2008; Pokusaeva et al. 2011; Anadón et al. 2015; Carlson and Slavin 2016). This prebiotic pool will surely expand further as research focusing on the role of prebiotics continues to stimulate the market, which is predicted to grow to approximately $10.55 billion by 2025 (Research and Markets 2019).

Cellooligosaccharides are composed of d-glucose monomers that are linked by a β-1,4-glycosidic bond and can, therefore, not be degraded by the human digestive enzymes. They are getting increasing attention as low-caloric fibres and potential prebiotics, as well as additives in pharmaceutical products (Rojas 2016). The common production route for cellodextrins is the chemical degradation of cellulose, the most abundant organic polymer on Earth (Klemm et al. 2005). Enzymatic alternatives include hydrolysis by cellulases (Kobayashi et al. 1991; Horn et al. 2012) or synthesis by phosphorylases (Luley-Goedl and Nidetzky 2010; Desmet and Soetaert 2012; Nakai et al. 2013). Regardless of the production method, the result typically is a mixture of carbohydrate chains with a varying degree of polymerisation (DP) (Mano et al. 2018).

**Fig. 1:**
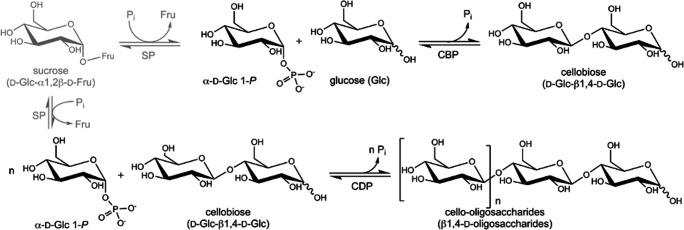
Reaction scheme of cellobiose (CBP) and cellodextrin phosphorylase (CDP). The in situ production of the high-energy intermediate α-d-glucose 1-phosphate (αG1-*P*) from sucrose is depicted in grey

The disaccharide cellobiose has already been produced in large amounts by the combined action of sucrose phosphorylase (SP; EC 2.4.1.7) and cellobiose phosphorylase (CBP; EC 2.4.1.20) (Koch et al. 2016) (Fig. 1). In this two-step, one-pot reaction, α-d-glucose 1-phosphate (αG1-*P*) serves as a high-energy intermediate that is continuously being regenerated in situ from sucrose (Koch et al. 2016) (Fig. 1). More recently, cellobiose elongation towards cellodextrins was achieved by adding cellodextrin phosphorylase (CDP; EC 2.4.1.49) to the reaction (Zhong et al. 2019). A maximal product concentration of 26 g/L was reported, and the resulting cellodextrin mixture consisted of DP2-6 at a ratio of 8/23/36/24/9 (Zhong et al. 2019). However, the production of the defined cellooligosaccharide with a high yield and purity remains challenging, which is unfortunate as cellotriose is the preferred substrate for *Bifidobacterium breve* UCC2003, a dominant probiotic bacterium in a healthy intestinal microbiota (Pokusaeva et al. 2011).

Here, we explored the possibility of engineering CDP and CBP for the defined synthesis of cellotriose. Although the synthetic reaction of CBP is the production of cellobiose from glucose and αG1-*P* (Fig. 1), a literature search revealed a previously created CBP variant that also showed activity on cellobiose as acceptor and was named OCP2 for its phosphorolytic activity on octyl β-cellobioside (De Groeve et al. 2010a). In this work, the products obtained by cellobiose elongation were determined, and semi-rational mutagenesis was applied, resulting in a CBP variant that provides the highest yield and purity of cellotriose reported to date. Furthermore, new insights into these enzymes’ specificities are also reported.

## Materials and methods

### Chemicals

All chemicals were obtained from Sigma-Aldrich (St. Louis, MO, USA) or Merck (Darmstadt, Germany), unless noted otherwise and were of the highest purity. Cellooligosaccharides of DP2-6 were obtained from Carbosynth (Compton, UK) or Megazyme (Bray, Ireland). The *Escherichia coli* BL21(DE3) strain was obtained from New England Biolabs (Beverly, USA).

### Gene cloning and transformation

The OCP2 variant of cellobiose phosphorylase from *Cellulomonas uda* (CuCBP_OCP2) (De Groeve et al. 2010a) was ordered as a synthetic gene from GeneArt and cloned in the isopropyl β-d-1-thio-galactopyranoside (IPTG) inducible pET21a plasmid with ampicillin resistance and N-terminal His_6_-tag. The cellodextrin phosphorylase from *Clostridium cellulosi* (CcCDP) was provided by the Austrian Center of Industrial Biotechnology, Graz, Austria, in their in-house designed constitutive plasmid (pC21e1) with ampicillin resistance and N-terminal His_6_-tag (Zhong et al. 2019). To avoid αG1-*P* degradation, the acid glucose 1-phosphatase gene (agp) was knocked-out of the *E. coli* BL21(DE3) strain using the recombinase/flippase gene disruption protocol (Datsenko and Wanner 2000). The final construct (*E. coli* BL21(DE3) agp^−^) was verified by sequencing (Macrogen) and used for transformation and heterologous expression of all enzymes.

### Site-directed mutagenesis

Site-directed mutations were introduced with a modified two-stage megaprimer-based whole-plasmid PCR method (Sanchis et al. 2008). The PCR mix contained 0.05 U/μl PfuUltra high-fidelity DNA polymerase (Stratagene, La Jolla, USA), 0.2 mM dNTP mix, 2 ng/μl template and 0.1 pmol/μl of each primer (Supplementary Table S1) in a total volume of 50 μl. The program started with an initial denaturation (3 min at 94 °C) followed by 5 cycles of denaturation for 30 s at 94 °C, annealing for 1 min at 55 °C and extension for 1 min/kb (size megaprimer) at 72 °C. The second stage consisted of 30 cycles of 10 s at 94 °C and extension for 1 min/kb (size entire plasmid) at 72 °C, followed by one final extension of 2 min at 72 °C. After digestion by *Dpn*I (Westburg, Leusden, The Netherlands), PCR products were purified by innuPREP PCRpure Kit (Analytik Jena, Jena, Germany). The plasmids were transformed in *E. coli* BL21(DE3) agp^−^ electrocompetent cells and subjected to nucleotide sequencing (Macrogen, Amsterdam, The Netherlands) after a plasmid mini-prep (Analytik Jena, Jena, Germany).

### Protein expression and cell lysis

Overnight cultures of CBP and CDP variants were inoculated (1% v/v) in 200 ml lysogeny broth (LB) containing 100 μg/ml ampicillin in 1-L shake flasks and incubated at 30 °C with continuous shaking at 200 rpm. Cultures were grown until the OD_600_ reached 0.6, and the enzyme production of CBP variants was induced by adding IPTG to a final concentration of 0.1 mM. Next, the culture was incubated overnight at 30 °C with continuous shaking at 200 rpm. The expression of CDP variants was achieved without induction due to a constitutive promoter. Cells were then collected by centrifugation, and the cell pellet was frozen at − 20 °C for at least 4 h. To obtain the enzyme, the cell pellet was slowly thawed on ice and resuspended in 10 ml lysis buffer composed out of 1 mg/ml of lysozyme (from chicken egg white), 100 μM phenylmethylsulfonyl fluoride (PMSF) and 50 mM 3-(N-morpholino)propanesulfonic acid (MOPS) at pH 7. Subsequently, the cells were sonicated three times for 3 min (Branson Sonifier 250, level 3, 50% duty cycle) or homogenised by glass beads (homogeniser FastPrep-24TM, MP Bio): 1.5 ml of glass beads were added to the lysate, and the mix was homogenised for 4 cycles of 30 s (4.0 m/s). Cell debris was removed by centrifugation at 4500 rpm for 30 min at 4 °C. Unless stated otherwise, the resulting crude cell extract was used for all reactions. Alternatively, the enzyme was further purified by a nickel-nitrilotriacetic acid (Ni-NTA) chromatography according to supplier’s protocol (MCLab, San Francisco, USA), after which the buffer was exchanged to 50 mM MOPS (pH 7) in a 30-kDa Amicon Ultra centricon using a 30 kDa cut-off (Merck Millipore, Darmstadt, Germany). The protein concentration was measured using the BCA Protein Assay kit (Pierce, Thermo Fisher Scientific, Waltham, MA, USA) with bovine serum albumin (BSA) as a standard.

### Cellooligosaccharide screening

The soluble cellooligosaccharides were separated and quantified by high-performance anion-exchange chromatography with a pulsed amperometric detection (HPAEC-PAD; Dionex ICS-3000 system, Thermo Fisher Scientific, Waltham, MA, USA), using a gradient method (0–11 min 100 mM NaOH, 11–20 min 90% 100 mM NaOH and 10% 100 mM NaOH/100 mM NaOAc, followed by 20–26 min 100% 100 mM NaOH for recalibration) and a flow rate of 0.5 ml/min. Analytical standards with different concentrations of αG1-*P*, glucose, cellobiose, cellotriose, cellotetraose and cellopentaose were used to detect and perform a quantitative analysis of cellodextrins. The results were visualised and plotted in SigmaPlot software (Systat Software Inc.).

### Reaction conditions and screening of mutants

To estimate whether CcCDP mutations result in a modified product profile, 3.5–4 mg/ml of protein crude cell extract was added into the reaction, and the outcome was evaluated after 4 h at 30 °C and pH 7. To follow cellooligosaccharide formation and substrate consumption with OCP2 variants, 6–7 mg/ml of crude cell extract was added into each enzymatic reaction, with varying substrate concentrations, and the outcome was followed over time at 40 °C and pH 7. Samples were inactivated by a 5-min incubation in a heat-block at 100 °C, and the denatured enzyme was removed by centrifugation for 5 min at 13500 rpm. Next, the samples were diluted 1500-fold in ultrapure water and analysed by HPAEC-PAD. The specific activity of the OCP2 variants was determined with respect to cellotriose production over time by using crude cell extracts, in 50 mM MOPS pH 7 at 40 °C. Cellotriose was quantified based on the HPAEC-PAD peak areas and using the analytical standard (0–50 μM).

### Enzyme kinetics

Unless stated otherwise, the activity was monitored in the synthetic direction by measuring the release of inorganic phosphate by the phosphomolybdate assay (Gawronski and Benson 2004; De Groeve et al. 2010b). One unit of the activity is defined as the amount of enzyme that generates 1 μmol of product per min under the used conditions. To determine the kinetic parameters of CcCDP variants, the reactions were performed with crude cell extracts in 100 mM 2-(N-morpholino)ethanesulfonic acid (MES) at pH 7 and monitored at 55 °C during 12 min. Samples of 100 μl were taken every 2 min for two acceptor concentrations (10 mM and 400 mM cellobiose with 50 mM αG1-*P* as a donor substrate). To determine the kinetic parameters of OCP2 and OCP2_M52R, the reactions were performed with purified enzymes in 50 mM MOPS at pH 7 and monitored at 40 °C for 12 min (with acceptor glucose or cellobiose) or 40 min (with acceptor cellotriose). Samples were taken every 2 or 5 min for each acceptor concentration (5–450 mM glucose; 5–250 mM cellobiose; 10–70 mM cellotriose, with 100 mM αG1-*P* as donor substrate). All samples were inactivated by the acidic conditions of the assay solution or by heating for 5 min at 100 °C and analysed with the phosphomolybdate assay to quantify the released phosphate. The kinetic parameters were calculated from Lineweaver-Burk linear regression (for CcCDP variants) and Michaelis-Menten non-linear regression (for OCP2 and OCP2_M52R) using SigmaPlot (Systat Software Inc.). The molecular weight of 111.6 kDa and 91.4 kDa was used to calculate the turnover number (*k*_cat_) for CcCDP and OCP2 variants, respectively.

### Ligand docking, homology modelling and sequence alignments

The homology models of the OCP2 and OCP2_M52R variants, as well as CcCDP, were generated with YASARA (Krieger and Spronk 2013; Land and Humble 2018) using default parameters. Template crystal structures for homology modelling were automatically selected by YASARA based on the highest scores. For OCP2 and OCP2_M52R, the enzyme structure of CBP from *Cellumonas uda* (CuCBP) served as a template: 3S4A (PDB entry). For CcCDP, the enzyme structure of CDP from *Clostridium thermocellum* (CtCDP) was used as a template: 5NZ7 (PDB entry). The CuCBP_OCP2 homology model and the crystal structures of CuCBP and CtCDP co-crystallised with cellobiose and cellotetraose, respectively, were aligned in YASARA by the built-in MUSTANG algorithm (Konagurthu et al. 2006; Krieger and Spronk 2013; Land and Humble 2018). Figures were made using PyMOL v2.0 (Schrödinger 2018 LLC, New York, USA). The reaction scheme of CBP and CDP was prepared in the ChemDraw program (PerkinElmer Informatics). The amino acid sequences were aligned in Clustal Omega webserver (Sievers and Higgins 2018) and manually curated based on the structural alignment.

### Sequence accession number

The sequences of CuCBP, CtCDP and CcCDP (UniProt identifiers: Q7WTR6, Q93HT8 and A0A078KL08, respectively) can be found in GenBank under accession numbers AAQ20920.1, BAB71818.1 and CDZ24361.1 for amino acid sequences, and AY343322.1, AB061316.1 and LM995447.1 for the nucleotide sequences, respectively.

## Results

### Engineering of cellodextrin phosphorylase

**Fig. 2:**
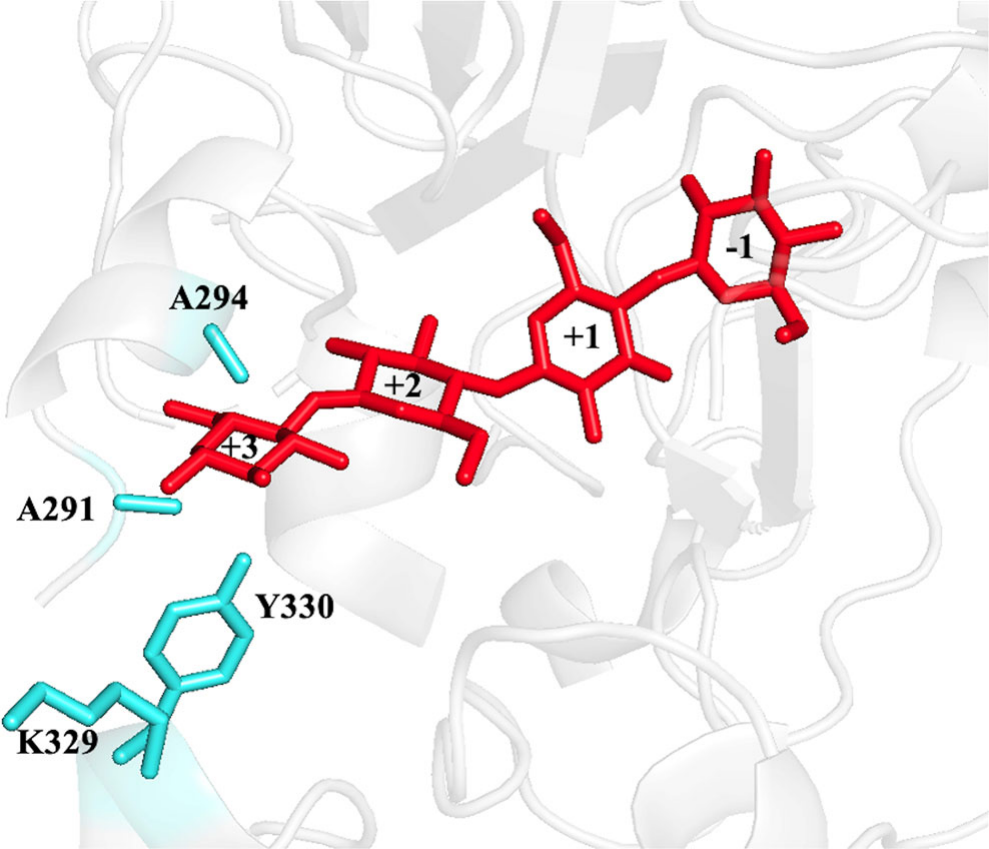
Docking of cellotetraose in subsite − 1/+ 3 of the CcCDP homology model. The residues targeted for site-directed mutagenesis are represented in light blue (Table S2)

The reported process for the controlled synthesis of water-soluble cellodextrins (DP2-6) was developed using the cellodextrin phosphorylase from *Clostridium cellulosi* (CcCDP) as biocatalyst (Zhong et al. 2019; Zhong and Nidetzky 2019). A strategy was now devised to shift this enzyme’s spectrum towards cellotriose as the main product. More specifically, it was envisaged that limiting substrate binding in subsite + 3 should stop chain elongation at DP3. To identify suitable positions for mutagenesis, a homology model of CcCDP was constructed, and cellotetraose was docked in subsites − 1 to + 3 (Fig. 2). Larger residues were introduced with the aim of blocking subsite + 3, whereas substitutions to smaller amino acids like alanine could potentially remove stabilizing interactions with cellotriose as an acceptor. In the end, nine single-point variants were prepared and recombinantly expressed in *E. coli* (Fig. 2, Supplementary Table S2). Unfortunately, the analysis of their reactions by HPAEC-PAD revealed that none of the mutations resulted in a significantly modified product profile (not shown) and almost all had a negative impact on the specific activity (Supplementary Table S2).

### Cellobiose phosphorylase as alternative biocatalyst

CBP and CDP both belong to the glycoside hydrolase 94 (GH94) enzyme family and, hence, share a number of structural features (Nakajima et al. 2017). However, CBP has a more closed active site that comprises only two subsites (− 1/+ 1) (Hai Tran et al. 2011). We, therefore, speculated that cellobiose phosphorylase might be a more suitable starting point for engineering towards cellotriose production. Previously engineered CBP variant from *Cellulomonas uda* (CuCBP_OCP2 or OCP2) that contained the five following mutations: N163D, N156D, T508I, E649G and N667A (Fig. 6c) exhibited synthetic activity on cellobiose (De Groeve et al. 2010a). Although the enzyme’s kinetics has been reported, the products obtained by cellobiose elongation have not yet been determined (De Groeve et al. 2010a).

Interestingly, an initial comparison of OCP2 with CcCDP not only demonstrated that OCP2 produces cellooligosaccharides, but also that it has a clear preference for cellotriose formation (Fig. 4a). A maximal conversion of 82% of cellobiose was observed, with cellotriose accounting for about 74% of the soluble cellodextrins (Table 1, Fig. 4a). Moreover, the cellotriose molar yield was around 2.5-fold higher with OCP2 compared to that of CcCDP (Table 1). As the reaction progresses, the enzyme starts to use the longer oligosaccharides as an acceptor, hence not consuming cellobiose beyond this point (Fig. 4a). Although the OCP2 variant did not exhibit a complete preference for cellotriose, its product profile certainly is much more promising than that of CcCDP.

**Table 1:** Optimal cellodextrin composition obtained with OCP2 and CcCDP (values are reported for the time points indicated with an arrow in Fig. 4a; DP3-cellotriose, DP4-cellotetraose, DP5-cellopentaose)

Enzyme	Cellobiose conversion (%)	DP3 molar yield (%)^a^	DP3 purity (%)^b^	DP4 molar yield (%)^a^	DP4 purity (%)^b^	DP5 molar yield (%)^a^	DP5 purity (%)^b^
CcCDP	69	25	28	16	19	16	18
OCP2	82	64	74	5	6	–	–

^a^The molar yield is calculated based on cellobiose consumption

^b^The purity is reported within the DP2-5 mixture

– not detected

### Further engineering of OCP2

**Fig. 3:**
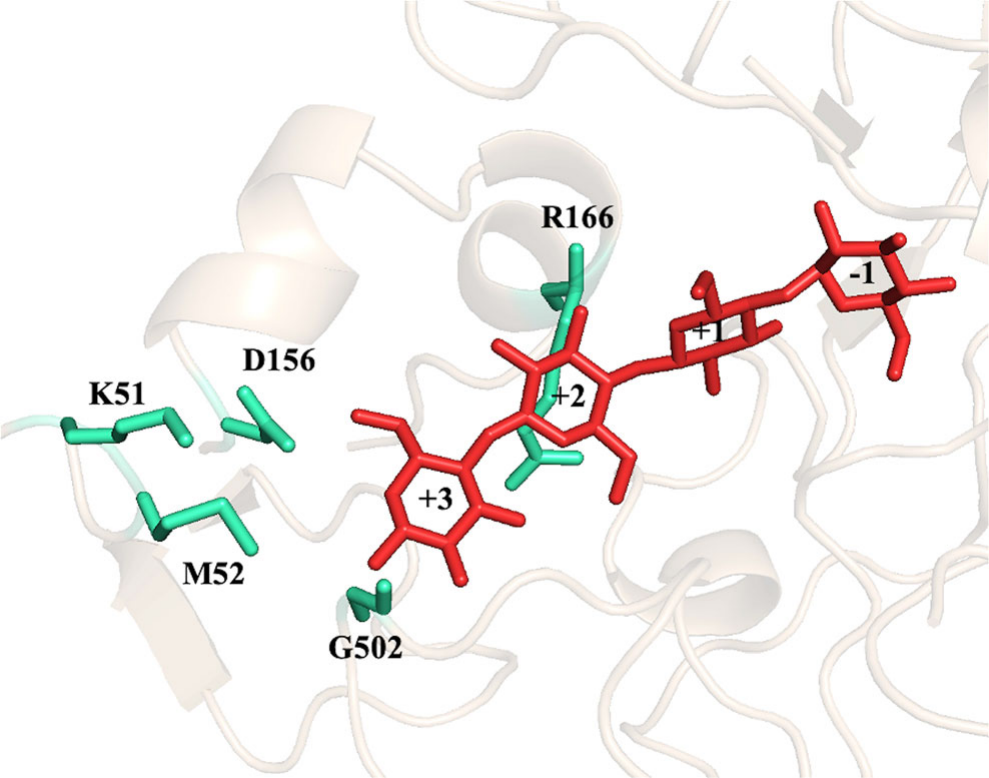
Active site of the CuCBP_OCP2 homology model with docked cellotetraose (in red). The residues targeted for site-directed mutagenesis are represented in green cyan (Table 2)

To identify target residues for further engineering of the OCP2 variant, a detailed analysis of its sequence and structure was conducted. A highly conserved aromatic residue at the + 2 subsite of CDP has been previously identified as specificity fingerprint that discriminates between CDP and CBP (Hai Tran et al. 2011). An alignment of CuCBP and CcCDP indeed shows that the latter contains an aromatic residue at this position (Y301) whereas the former contains an Arg residue (R166) (Fig. 3, Fig. 5). The corresponding substitution has now been introduced in OCP2 (R166Y) to evaluate the impact on cellotriose synthesis (Table 2). The mutation, however, resulted in a completely inactive enzyme (Table 2), highlighting the high evolutionary significance of this residue for CBP activity.

**Table 2:** Screening of different OCP2 variants (specific activity was measured during 4–5 h (1.5 h for OCP2_M52R) using crude cell extracts, 100 mM cellobiose and 400 mM αG1-*P*, at pH 7 and 40 °C; one unit of the activity is defined as the amount of the enzyme that released 1 μmol of cellotriose/min; DP3-cellotriose, DP4-cellotetraose)

Mutant	Specific activity (U/mg)^a^	Cellobiose conversion (%)^b^	DP3 purity (%)^c^	DP4 purity (%)^c^
OCP2	0.04	95	76	16
OCP2_D156W	Inactive	–	–	–
OCP2_D156Y	0.01	81	70	3
OCP2_D156R	0.03	97	77	18
OCP2_D156K	0.04 ± 0.02	99	69	27
OCP2_M52W	0.01	97	77	17
OCP2_M52R	0.16	89	82	3
OCP2_K51R	0.07 ± 0.01	93	74	15
OCP2_G502N	0.02	90	65	12
OCP2_R166Y	Inactive	–	–	–

^a^Standard deviations are calculated based on triplicates and were ≤ 10%, unless stated otherwise

^b^All samples were taken after 22 h when approximately all variants reached the maximal cellobiose conversion, except for OCP2_M52R that reached the maximal conversion after 4 h

^c^The purity is reported within the DP2-5 mixture

– not determined

Docking of cellotetraose in the active site, the OCP2 model highlighted M52, D156, K51 and G502 as the most interesting target residues for further mutagenesis (Fig. 3). Eight different variants were thus created, of which M52R turned out to be the best one. Indeed, this mutation resulted in the highest cellotriose formation (82%) and the lowest cellotetraose accumulation (3%), as well as a fourfold increase in specific activity (from 0.04 to 0.16 U mg^−1^) (Table 2). Since all other variants displayed features that were overall not better than those of the template enzyme, only OCP2_M52R was characterised in detail.

### Detailed characterisation of OCP2_M52R

**Fig. 4:**
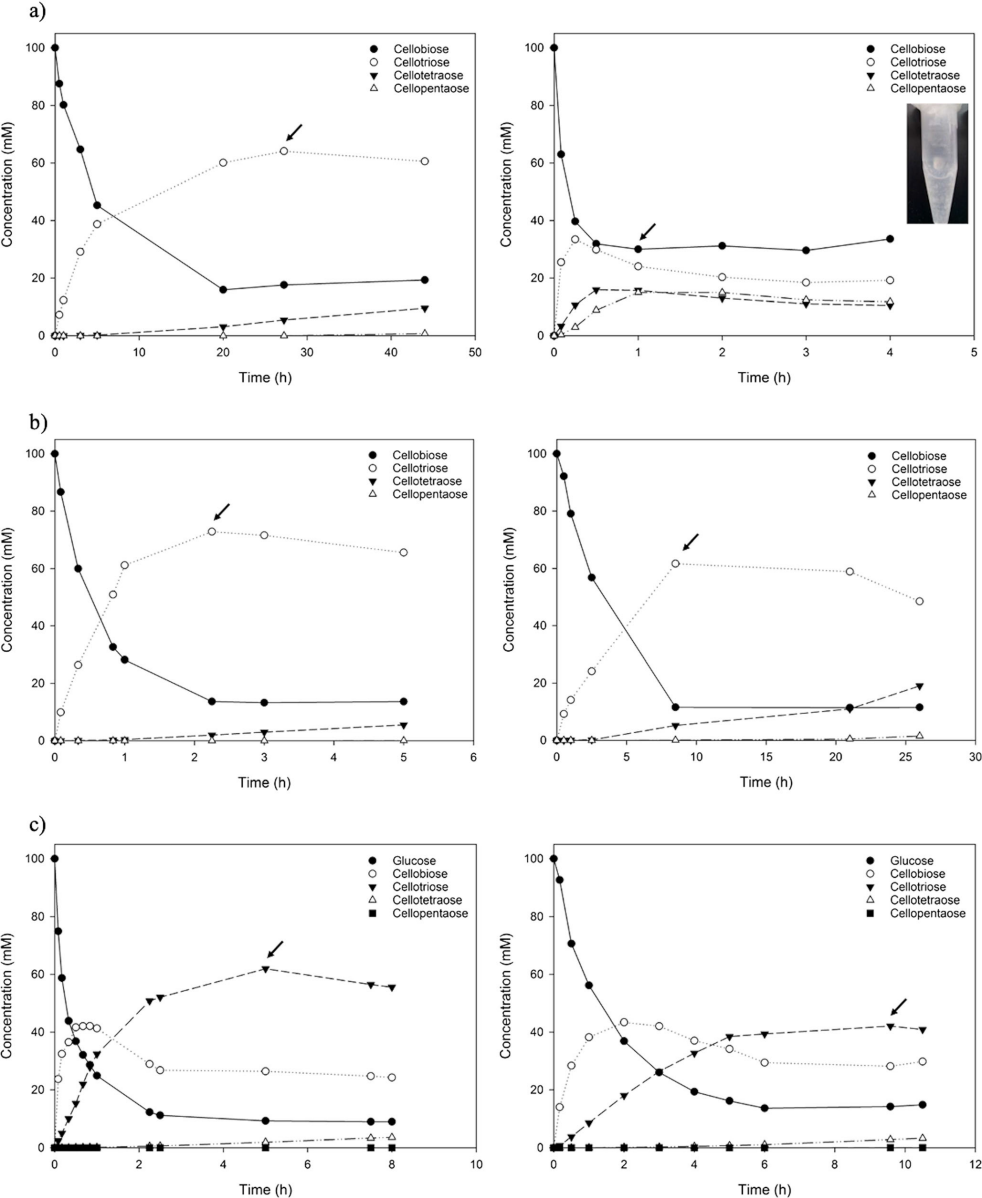
Cellodextrin formation with different enzyme variants at pH 7 and 40 °C. The arrow indicates the time point for which concentrations are listed in the tables. **a** OCP2 (left) and CcCDP (right), using 100 mM cellobiose and 200 mM αG1-*P* (Table 1). **b** OCP2_M52R (left) and OCP2 (right), using 100 mM cellobiose and 400 mM αG1-*P* (Table 3). **c** OCP2_M52R (left) and OCP2 (right), using 100 mM glucose and 400 mM αG1-*P* (Table 3). The data are from single representative time-course experiment but are within ≤ 10% for replicates (*N* ≤ 3)

To further elucidate the differences between OCP2 and OCP2_M52R concerning cellotriose synthesis, reactions with a 0.25 M ratio of cellobiose/αG1-*P* were followed over time (Fig. 4b, Table 3). An excess of αG1-*P* was used to push the enzymes towards the synthesis of longer cellodextrins, thus challenging OCP2_M52R’s preference towards cellotriose production. Moreover, it has been previously reported that the molar ratio of acceptor/αG1-*P* was the main variable affecting the produced DPs with both CBP and CDP (Zhong et al. 2019). When 200 mM αG1-*P* was used, the production of soluble cellodextrins from glucose required this ratio to be 0.25 or lower (Zhong et al. 2019).

**Table 3:** Optimal cellodextrin composition obtained with OCP2 and OCP2_M52R (values are reported for the time points indicated with an arrow in Fig. 4b, c; DP2-cellobiose, DP3-cellotriose, DP4-cellotetraose, DP5-cellopentaose)

Enzyme	Acceptor	Conversion (%)	DP2 molar yield (%)^a^	DP2 purity (%)^b^	DP3 molar yield (%)^a^	DP3 purity (%)^b^	DP4 molar yield (%)^a^	DP4 purity (%)^b^	DP5 molar yield (%)^a^	DP5 purity (%)^b^
OCP2_M52R	Cellobiose	86	–	–	73	82	2	2	–	–
OCP2	Cellobiose	88	–	–	62	79	5	7	0.05	0.06
OCP2_M52R	Glucose	91	26	27	62	62	2	2	–	–
OCP2	Glucose	86	28	32	42	48	3	3	–	–

^a^The molar yield is calculated based on acceptor consumption

^b^The purity is reported within the DP1–5 (with glucose as acceptor) or DP2-5 (with cellobiose as acceptor)

– not detected

The maximal conversion of cellobiose to cellodextrins was similar with both variants, namely about 87% (Fig. 4b, Table 3). The reaction with OCP2_M52R, however, resulted in a 2.5-fold lower yield of cellotetraose compared to OCP2 and slightly higher yield (73% vs 62%) and purity (82% vs 79%) of cellotriose, thus confirming the previous findings (Table 2). Both variants were further evaluated concerning the products synthesised from glucose, which is considerably cheaper than cellobiose as acceptor (Fig. 4b, Table 3). The results again showed that OCP2_M52R performs better with the purity of cellotriose, reaching maximal 62%, contrary to the 48% achieved with OCP2 (Table 3). Although the maximal conversion of cellobiose was similar with both variants, OCP2_M52R was slightly more efficient in converting glucose (91%) compared to OCP2 (86%) (Table 3).

To further elucidate the differences between OCP2 and its variant M52R, a detailed kinetic characterisation was conducted (Table 4). The results showed that the latter has a fourfold lower Michaelis-Menten constant (*K*_m_) for cellobiose, while the *K*_m_ for cellotriose was not significantly affected (Table 4). Both enzymes exhibited a higher affinity for cellobiose than for glucose (Table 4). With cellobiose as an acceptor, the catalytic efficiency of OCP2_M52R was around sevenfold higher than that of OCP2. When glucose and cellotriose were used as acceptors, the catalytic efficiency was similar in both variants.

**Table 4:** Kinetic parameters on different acceptors (using 100 mM αG1-P, at pH 7 and 40 °C)

Enzyme	Glucose			Cellobiose			Cellotriose		
	*K*_m_ (mM)	*k*_cat_ (s^−1^)	*k*_cat_/*K*_m_ (M^−1^ s^−1^)	*K*_m_ (mM)	*k*_cat_ (s^−1^)	*k*_cat_/*K*_m_ (M^−1^ s^−1^)	*K*_m_ (mM)	*k*_cat_ (s^−1^)	*k*_cat_/*K*_m_ (M^−1^ s^−1^)
OCP2	19.0 ± 2.1	1.9 ± 0.0	100.0 ± 23.8	10.2 ± 0.8	1.2 ± 0.0	117.6 ± 21.1	5.3 ± 1.7	0.06 ± 0.003	11.3 ± 1.7
OCP2_M52R	43.8 ± 4.0	4.7 ± 0.1	107.3 ± 25.0	2.3 ± 0.6	2.0 ± 0.1	869.6 ± 166.7	5.4 ± 1.6	0.05 ± 0.003	9.2 ± 1.8

## Discussion

An efficient process has recently been reported for the controlled synthesis of soluble cellooligosaccharides (DP2-6) from glucose in a combined enzymatic reaction with SP, CBP and CDP (Zhong et al. 2019). However, cellotriose accounted for only 23% of the product mixture, which is consistent with the results obtained here with just CDP as biocatalyst (cellotriose purity of 28%). In an attempt to disrupt subsite + 3 of CcCDP and hamper further chain elongation, nine single-point mutants were created. As none of these showed a modified product profile, it was concluded that the active site cleft of CDP is most likely too spacious to interfere with acceptor binding through a simple amino acid substitution. Extensive remodelling of the active site (e.g., by loop insertions) could be attempted, but that typically is a very challenging undertaking with a minimal chance of success. A better starting point was found in the cellobiose phosphorylase variant OCP2, which was previously shown to use cellobiose as acceptor but had not yet been characterised in detail. This biocatalyst offered a 2.5-fold higher yield (64% vs 25%) and purity (74% vs 28%) of cellotriose compared to CcCDP. It should be noted that a fraction of the acceptor substrate is converted to longer cellooligosaccharides that precipitate out of the reaction mixture due to their low solubility.

**Fig. 5:**
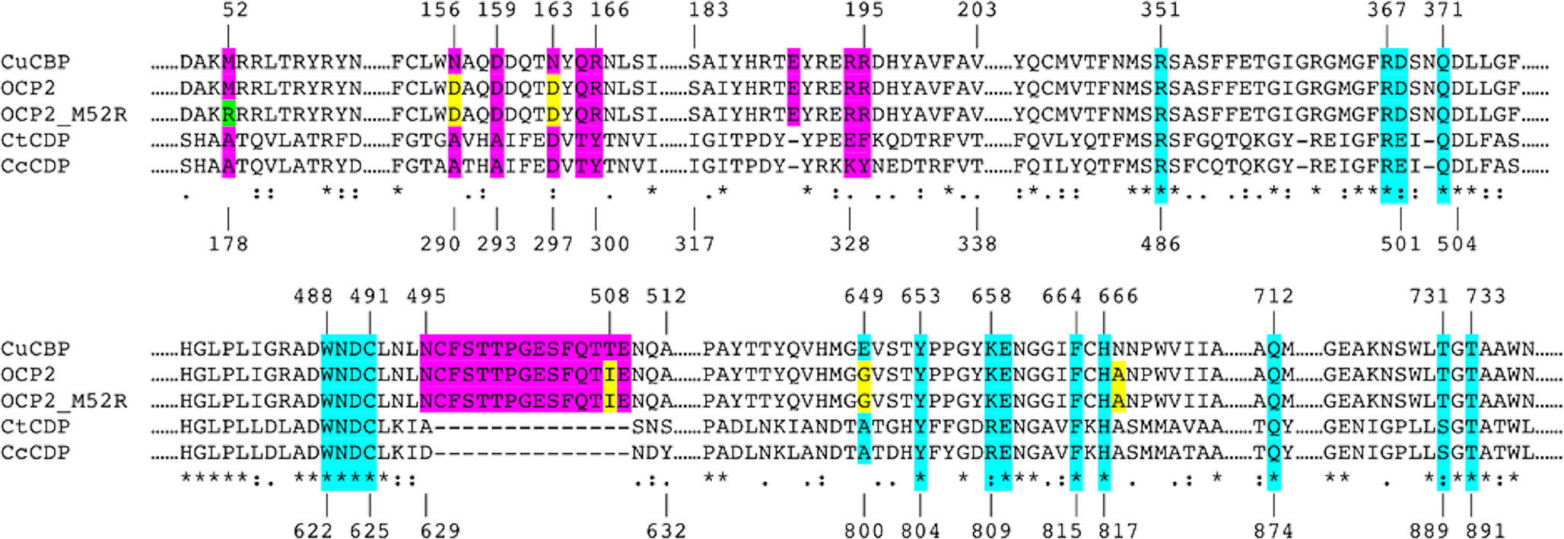
Multiple sequence alignment of selected GH94 enzymes. Highly conserved residues that build up subsites − 1/+ 1 are represented in cyan blue. The residues that build up subsites + 2/+ 3 in CDPs are represented in purple. The five mutations in OCP2 are represented in yellow, while the newly introduced M52R mutation is represented in green

**Fig. 6:**
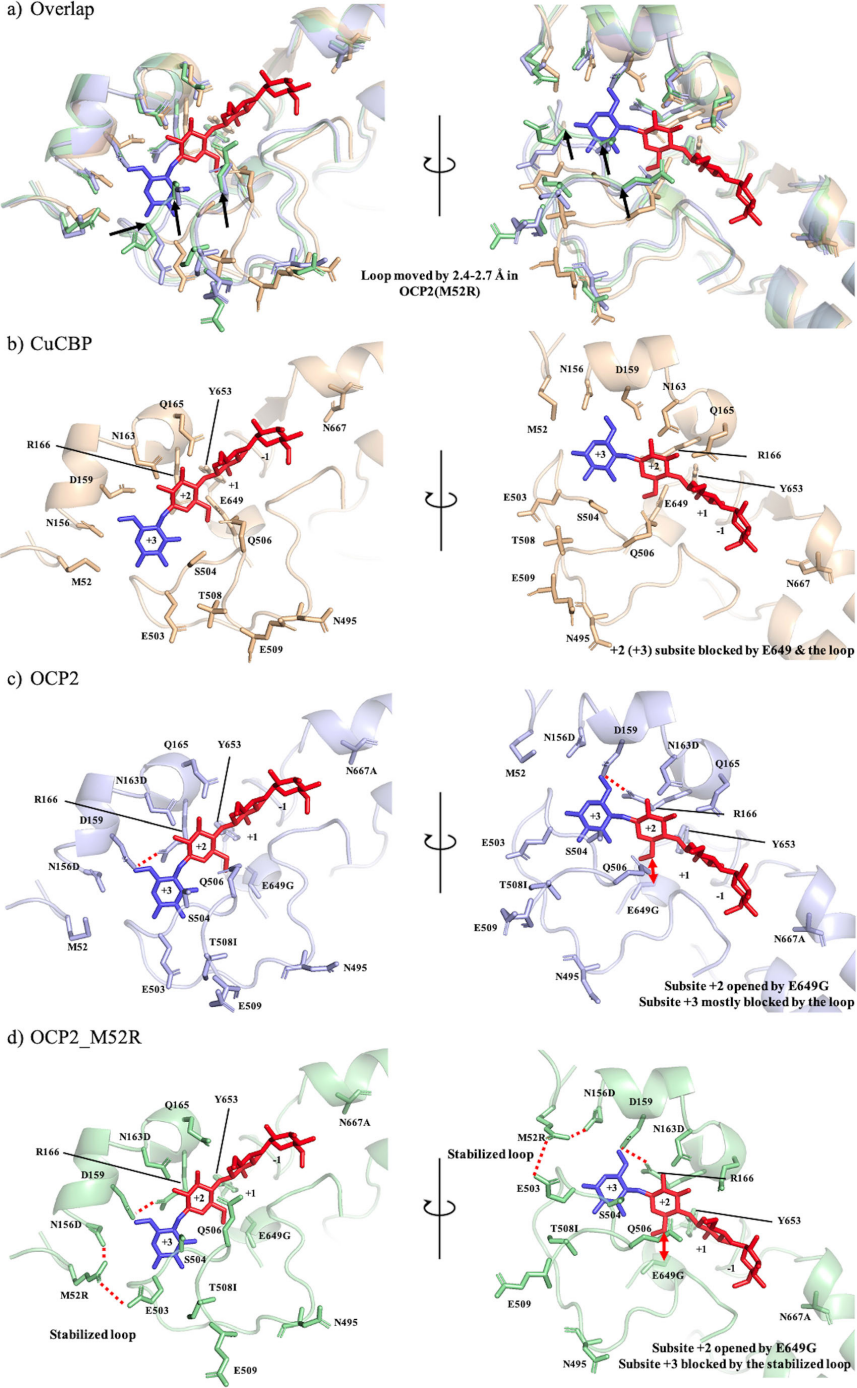
Several homology models with docked cellotetraose coloured in dark blue (+3 subsite) and red (− 1, + 1 and + 2 subsites). **a** OCP2(M52R) superimposed with CuCBP and the impact of T508I indicated with arrows. **b** CuCBP with an active site that is closed for larger acceptors. **c** OCP2 in which the structural changes imposed by T508I and E649G are shown. **d** OCP2_M52R in which the loop moved inwards to further block the subsite +3

To provide an explanation for the OCP2 catalytic behaviour compared to wild-type CBP and CDP, their structures and sequences have been analysed in more detail. The five OCP2 mutations are all located in (close vicinity of) the catalytic cleft (Fig. 6c). Previous experiments have demonstrated that only two mutations, T508I and N667A, are needed to introduce synthetic activity on cellobiose (De Groeve et al. 2010a). Residue N667 is located at subsite − 1, and its substitution with Ala introduces the residue that is present in CDPs, thus making CBP more similar to the elongating enzymes (Fig. 5). In turn, T508 is part of a loop (N495-E509) that is present in CBPs but absent in CDPs, and blocks subsites + 2/+3 (Fig. 5, Fig. 6). Its substitution with Ile seems to move the loop closer to the + 3 subsite and block it even more (Fig. 6a). At the same time, the side chain of Q506 is turned inwards to form hydrogen bonds with the glucose ring in subsite +2, thus improving the binding of cellobiose as acceptor (Fig. 6a–c). Although less crucial, two of the additional mutations make the active site of OCP2 more similar to that of CDP. On the one hand, the E649G substitution could mimic the Ala present in CDP to create more space for cellobiose in subsite + 2 (Fig. 6c). On the other hand, the N163D substitution introduces the Asp residue present in CDP and further enhances the similarity of its subsite + 2 (Fig. 5).

Despite the obvious preference towards cellotriose synthesis, the OCP2 variant demonstrated a very low activity (0.04 U/mg) and low affinity (*K*_m_ of 10.2 mM) for the acceptor cellobiose. Further engineering efforts yielded the OCP2_M52R variant with a fourfold lower *K*_m_ for cellobiose and fourfold higher activity. Furthermore, this variant also offers a higher cellotriose yield (73 instead of 62%) and a drop in further elongation (from 5 to 2%), thus giving an improved product purity compared to the OCP2 starting point. Remarkably, both variants exhibit a higher affinity for cellobiose compared to glucose, which is unusual, considering that glucose is the natural acceptor for CBP. However, OCP2_M52R has a twofold lower *K*_m_ for cellobiose compared to cellotriose, while OCP2 exhibits the opposite trend, and this could be one of the main reasons for the improved performance in cellotriose production. In the homology model of OCP2_M52R, the newly introduced Arg can be seen blocking subsite +3 through interactions with Glu503 and Asp156 (Fig. 6d).

Based on the conditions considered in this work, scale-up of the process to 27 L with ~ 190 g of OCP2_M52R crude cell extract would yield 1 kg of cellotriose starting from 0.92 kg of cellobiose and 4.3 kg of αG1-*P* (disodium salt tetrahydrate). The phosphate species could then be removed with an ion-exchange resin (Van Der Borght et al. 2010) to obtain cellotriose at a purity of 82%. Since the enzyme variant is derived from CBP, it is also able to use glucose as acceptor. In that case, 0.58 kg of glucose would be needed in a volume of 32 L, with ~ 225 g of the enzyme crude cell extract. The remaining acceptor could afterwards be removed by selective fermentation (Yoon et al. 2003) to obtain cellotriose at a purity of 62%. Furthermore, if αG1-*P* is to be regenerated through coupling with sucrose phosphorylase, the fructose released from sucrose could be converted to glucose as the required acceptor substrate with the help of glucose isomerase. Overall, the improved enzyme variant reported here should be a crucial step in enabling further research on and commercial exploitation of cellotriose.

## Electronic supplementary material

ESM 1 (PDF 156 kb)

## Data Availability

Data obtained in the current study are available from the DOI 10.5281/zenodo.3932176.
