# Supplementary material for: Engineering of cellobiose phosphorylase for the defined synthesis of cellotriose
Source: Appl Microbiol Biotechnol. 2020 Aug 17;104(19):8327–37. doi: 10.1007/s00253-020-10820-8 (PMC7471185; doi:10.1007/s00253-020-10820-8)
Supplement: Supplementary file 1 — (PDF 156 kb) [file 253_2020_10820_MOESM1_ESM.pdf]

Supplementary Material

Applied Microbiology and Biotechnology

Engineering of cellobiose phosphorylase for the defined synthesis of cellotriose

Zorica Ubiparip[1], David Sáez Moreno[1], Koen Beerens[1], Tom Desmet[1*]

[1] Centre for Synthetic Biology (CSB), Department of Biotechnology, Ghent University, Coupure Links 653, 9000 Ghent, Belgium

[*] **Corresponding author:**
Tom Desmet, Centre for Synthetic Biology
E-mail: tom.desmet@ugent.be
Website: www.biocatalysis.ugent.be

Table S1. Primers used for enzyme engineering; mutations are underlined

Construct	Template	Primer Sequences (5'-3')
A291R	CcCDP wild-type	CATGTTTGGTACCGCGAGGACCCACGCGATCTTT
A291F	CcCDP wild-type	GGCATGTTTGGTACCGCGTTCACCCACGCGATCTTTGA
A294H	CcCDP wild-type	GGTACCGCGGCGACCCACTATATCTTTGAAGATGTGACCT
A294F	CcCDP wild-type	GTACCGCGGCGACCCACTTCATCTTTGAAGATGTGACC
Y330A	CcCDP wild-type	TACCCCGGATTACTATCGTAAGAAAGCCAACGAAGACACCC
Y330R	CcCDP wild-type	TACCCCGGATTACTATCGTAAGAAACGCAACGAAGACACCC
K329R	CcCDP wild-type	CGGATTACTATCGTAAGAGATACAACGAAGACACCCG
K329A	CcCDP wild-type	CCCGGATTACTATCGTAAGGCATACAACGAAGACACCCGT
K329W	CcCDP wild-type	ACCCCGGATTACTATCGTAAGTGGTACAACGAAGACACCCGTTTC
CcCDP_RV1	CcCDP wild-type	CGAAATAACGCAGCAGCGCTTCCAGCTCTTTCG
CcCDP_RV2	CcCDP wild-type	CGTTGCTGGTCCACTCAAACAGCAGGTTCTTC
K51R	CuCBP_OCP2	TTTTACCGCGATGCAAGGATGCGTCGGTTGACC
M52W	CuCBP_OCP2	TACCGCGATGCAAAGTGGCGTCGGTTGACCCG
M52R	CuCBP_OCP2	GCTTTTACCGCGATGCAAAGAGGCGTCGGTTGA
D156W	CuCBP_OCP2	TTCGTGGAGTTTTGCTTATGGTGGGCCCAAGATGATCAGACTGAC
D156K	CuCBP_OCP2	CGTGGAGTTTTGCTTATGGAAGGCCCAAGATGATCAGACTG
D156R	CuCBP_OCP2	GTGGAGTTTTGCTTATGGCGCGCCCAAGATGATCAGAC
D156Y	CuCBP_OCP2	CGTGGAGTTTTGCTTATGGTATGCCCAAGATGATCAGACTG
G502N	CuCBP_OCP2	AACTGCTTTTCCACCACGCCGAATGAAAGCTTTCAAACCATCGAA
R166Y	CuCBP_OCP2	CAAGATGATCAGACTGACTACCAATATAACCTGAGTATCGGCGAAGTG
CuCBP_OCP2_RV1	CuCBP_OCP2	TTGCGCTGCCATGAGGACTCTCCTGTTCTAC
CuCBP_OCP2_RV2	CuCBP_OCP2	AGACGCTGACCGACTCATGTTAAACGTGAC
CuCBP_OCP2_RV3	CuCBP_OCP2	GGTACGTAGATACGCCGCCCATGTGGAC

Table S2. Kinetic parameters towards cellobiose as acceptor[a]

Enzyme	V_{max} (U/mg)	k_{cat} (s^{-1})	K_m (mM)	k_{cat}/K_m (M^{-1} s^{-1})
CcCDP (wild-type)	10.4	19.3	16.8	1149
CcCDP_A294F	0.35	0.6	1.3	461
CcCDP_A294H	0.17	0.3	2.2	136
CcCDP_A291R	3.9	7.2	27.2	265
CcCDP_A291F	5.1	9.5	18.3	519
CcCDP_K329R	0.5	0.9	4.3	209
CcCDP_K329A	3.7	6.9	5.7	1210
CcCDP_K329W	0.5	0.9	4.2	214
CcCDP_Y330A	0.6	1.1	2.5	44
CcCDP_Y330R	0.7	1.3	10.5	124

[a] Using 50 mM αG1-*P* as co-substrate in 100 mM MES buffer, at pH 7 and 55 °C
